# Status of hypertension screening in the Korea National General Health Screening Program: a questionnaire survey on 210 screening centers in two metropolitan areas

**DOI:** 10.1186/s40885-017-0075-z

**Published:** 2017-12-05

**Authors:** Seung Won Lee, Hae-Young Lee, Sang Hyun Ihm, Sung Ha Park, Tae Hyun Kim, Hyeon Chang Kim

**Affiliations:** 1 Cardiovascular and Metabolic Diseases Etiology Research Center, Yonsei University College of Medicine, Seoul, South Korea; 2 Department of Preventive Medicine, Yonsei University College of Medicine, Seoul, 03722 South Korea; 3 Department of internal medicine, Seoul National University College of Medicine, Seoul, South Korea; 4 Division of Cardiology, College of Medicine, Catholic University of Korea, Seoul, South Korea; 5 Department of internal medicine, Yonsei University College of Medicine, Seoul, South Korea; 6 Graduate School of Public Health, Yonsei University, Seoul, South Korea

**Keywords:** Hypertension, Blood pressure, Screening, Sphygmomanometer, Quality control

## Abstract

**Background:**

The purpose of this survey was to evaluate the performance of hypertension screening in medical institutions conducting the national general health screening program of the Republic of Korea.

**Methods:**

We contacted 700 medical institutions of Seoul and Incheon areas which performed the national general health screening program in 2016, and 210 of them completed telephone survey. The questions asked in the survey include equipment, environment, personnel and quality control procedures for blood pressure (BP) measurement, and interpretation of the measurements.

**Results:**

A majority of the responding screening centers used oscilloscope sphygmomanometers (51.9%), had only one-sized cuff (65.2%), and measured BP in open space (54.3%). BP levels were measured mainly by nurses (62.0%) and doctors (25.0%), after a 1 to10 minutes (84.9%) of resting period. A 75.2% of screening centers regularly calibrated sphygmomanometers, 81.4% had a manual for BP measurement, and 59.0% had a training program. A 80.0% of respondents answered that they used averages of multiple BP measurements to determine an individual’s BP level, and 82.9% answered that criteria for hypertension was systolic BP ≥140 mmHg and/or diastolic BP ≥ 90 mmHg. If a screening finds an individual with hypertension, 82.9% of centers recommend revisiting for a second BP measurement rather than start medication immediately.

**Conclusion:**

In most medical institutions performing general health screening program, certified medical personnel measure BP and interpret the results according to established protocols. However, there is room for improvement in the equipment, environment and quality control procedures for BP measurement.

## Background

Hypertension, the most important modifiable risk factor for cardiovascular diseases, is a major health burden and the leading cause of death in the worldwide [1–4]. Previous studies reported that the reduction of highly or moderately elevated BP levels results in a decrease in stroke and myocardial infarction rates [5, 6]. Every 10 mmHg increase in systolic blood pressure (BP) or diastolic BP is estimated to double the risk of death from ischemic heart disease and stroke [7].

In Korea, diseases of circulatory system including coronary heart disease and stroke accounted for 22% of the entire death in 2015 [8]. The number of people who visited the hospital for chronic diseases in 2015 was 14.39 million, with the highest number of hypertension (5.71 million) among them in Korea. Prevalence of hypertension was estimated to about 35.1% for male, and 29.1% for female among Korean adults aged 30 years and above in 2015 [9]. According to the Korean Nation Health Survey in 2001, the age-adjusted prevalence of hypertension was 22.9% (26.9% in men and 20.5% in women) [10]. Especially, hypertension is a common disease among Korean elderly population aged 65 years or older considering that its prevalence increased between 2007 and 2011 from 49.3% to 58.4% in men and from 61.8% to 68.9% in women [11]. Total medical cost for hypertension was estimated to 2850 billion Korean won (KRW) which accounts for 13.4% of medical cost due to all chronic disorders [12]. This means that controlling hypertension is crucial to reducing the overall burden of disease in society and improving quality of life. Therefore, many countries carry out national health screening and emphasize the importance of measuring BP. The benefits of screening for hypertension are well established [13–15]. However, although hypertension screening is a very important for preventing of hypertension, there have been few studies on hypertension screening in Korea. Thus, we evaluated the performance of hypertension screening and interpretation of BP measurements in medical institutions performing the national general health screening program of the Republic of Korea.

## Methods

This descriptive survey aims to collect information on protocol of BP measurement, setting condition of measurement place, diagnosis of hypertension, and criteria of medicine prescription. First, we made a multiple-choice questionnaire to conduct a survey using telephone. The survey was conducted by trained telephone interviewers for February and March of 2016. A total of 5435 medical institutions, including clinics, general hospital, long-term care hospital, hospitals, and health center, were able to conduct surveys in 2016. We contacted 700 medical institutions of Seoul and Incheon areas which performed the national general health screening program in 2016, and 210 of them completed telephone survey. We made questionnaire items based on hypertension screening protocol of Korea National Health Insurance [16] with reviewing seventh and eighth report of the Joint National Committee (JNC) [17, 18], and 2013 guidelines on hypertension of the European Society of Hypertension and the European Society of Cardiology (ESH/ESC) [19]. According to protocol for BP measurement in national health screening program, all examinees had rested for at least 5 min in a seated position prior to first measurement. BP should be measured by the auscultation method using a stethoscope or using an oscilloscopic automatic sphygmomanometer in a quiet environment. The calibration of devices should be checked day-to-day. A cuff with an appropriately sized bladder should be used. The standard bladder for adults is 12 cm wide and 26 cm long. The use of a bladder with a width of at least 40% of the circumference of the arm and a length of 80% to 100% of the circumference of the arm is recommended. Examinees are recommended to avoid smoking, alcohol, or caffeine before measurement. We made the questionnaire items reflecting these protocols.

## Results

A 210 responding medical institutions includes 154 clinics, 38 hospitals, 15 general hospitals, 2 long-term care hospitals, and 1 health check-up center (Table 1). Most screening centers (51.9%) used oscilloscope sphygmomanometer to measure BP, and the percentage of medical institutions using only mercury sphygmomanometers was 44%. A 75.2% of medical institutions regularly check whether sphygmomanometers operate normally. A 65.2% of the medical institutions had only one cuff size, and 40.5% said they use a thermometer. A majority of the screening centers used open space (54.3%) to measure BP and 28.6% of the medical institutions used an independent place for BP measurement only. People who measure BP were mainly the nurse (62%) and the doctor (25%).

**Table 1:** Physical and human resources related to blood pressure measurement

Classification	n	(%)
Type of medical institution		
Clinic	154	(73.3)
Hospital	38	(18.1)
General hospital	15	(7.1)
Long-term care hospital	2	(1.0)
Health check-up center	1	(0.5)
Type of sphygmomanometer		
Oscilloscope	109	(51.9)
Mercury	44	(21.0)
Aneroid	1	(0.5)
Mercury and oscilloscope	50	(23.8)
Aneroid and oscilloscope	5	(2.4)
Mercury, aneroid and oscilloscope	1	(0.5)
Cuff size		
One	137	(65.2)
Two	66	(90.4)
Three or more	7	(9.6)
Existence of a thermometer		
Yes	85	(40.5)
No	125	(59.5)
Sphygmomanometer calibration before measurement		
Yes	158	(75.2)
Daily	75	(47.5)
Weekly	43	(27.2)
Monthly	39	(24.7)
No response	1	(0.6)
No	52	(24.8)
Place of blood pressure measurement		
Open space (e.g. waiting room, reception desk and lobby)	114	(54.3)
Separate room for measurement of blood pressure	60	(28.6)
Separate room for measurements of blood pressure and other examinations	18	(8.6)
Open space or separate room	16	(7.6)
No specified place	1	(0.5)
No response	1	(0.5)
Person who measure blood pressure		
Nurse	130	(62.0)
Physician	53	(25.0)
Medical technician	14	(7.0)
Nurse’s aide	10	(5.0)
Administrative staff	2	(1.0)
Examinee (automated measurement)	1	(0.0)

BP was measured twice or more in 59.5% of medical institutions, and 35.2% of medical institution measured BP only one time (Table 2). A 18.6% of the medical institutions did not have manual to measure BP and 40.5% did not perform the BP measurement training. A majority of the medical institutions (93.8%) did not measure the arm circumference to select the appropriate cuff size. A 94.8% of medical institutions said they would let people take a rest before measuring BP, and 5% said they did not give people time to relax.

**Table 2:** Methods of measuring blood pressure and related resources

	Number	Percent
Number of measurement		
Once	74	(35.2)
Twice	109	(51.9)
Triple or more	16	(7.6)
Etc.	11	(4.2)
Existence of a manual		
Yes	171	(81.4)
No	39	(18.6)
Existence of an operator training program		
Yes	124	(59.0)
No	85	(40.5)
No response	1	(0.5)
Measurement of arm circumference		
Yes	12	(5.7)
No	197	(93.8)
No response	1	(0.5)
Resting time before measurement		
Yes	199	(94.8)
1–10 min	169	(84.9)
11–20 min	22	(10.5)
21–30 min	8	(4.0)
No	11	(5.2)

In the majority of medical institutions (82.9%), the definition of hypertension was systolic BP ≥ 140 mmHg or diastolic BP ≥ 90 mmHg (Table 3). A 56.2% of the medical institutions applied different diagnostic criteria for hypertension according to sex, age, and accompanying diseases. When an examinee has high blood pressure at first visit, the most common strategy is recommendation to revisit after 1 to 2 weeks to measure BP again.

**Table 3:** The criteria related to hypertension by medical institutions participated in the survey

	Number	Percent
Use the average of multiple measurements to determine an individual’s BP level		
Yes	168	(80.0)
No	42	(20.0)
Definition of hypertension for people younger than 65 years		
SBP ≥ 130 mmHg or DBP ≥ 90 mmHg	21	(10.0)
SBP ≥ 140 mmHg or DBP ≥ 90 mmHg	174	(82.9)
SBP ≥ 160 mmHg or DBP ≥ 90 mmHg	8	(3.8)
No response	7	(3.3)
Use different hypertension criteria according to sex, age, and accompanying disease		
Yes	118	(56.2)
No	92	(43.8)
Definition of hypertension for people older than 65 years (*n* = 118)		
SBP ≥ 130 mmHg or DBP ≥ 90 mmHg	9	(7.6)
SBP ≥ 140 mmHg or DBP ≥ 90 mmHg	83	(70.3)
SBP ≥ 160 mmHg or DBP ≥ 90 mmHg	20	(16.9)
No response	6	(5.1)
Recommendation for people with high blood pressure		
Revisit and measure blood pressure again	174	(82.9)
24-h ambulatory blood pressure measurement	17	(8.1)
Prescription anti-hypertensive drug	16	(7.6)
No response	3	(1.4)

Table 4 is as to which levels of the patient’s BP a doctor decided to treatment. The majority of medical institutions prescribe medication when an examinee has at least above systolic BP of 150 mmHg considering remeasured BP together (Table 4). When the examinee had a systolic BP of 140 mmHg and a diastolic BP of 80 mmHg, 57.1% of medical institutions did not recommend the use of anti-hypertensive medication, and 39.5% of medical institutions recommended medication. When the examinee had a systolic BP of 150 mmHg and a diastolic BP of 95 mmHg, 16.7% of the medical institutions did not recommend the use of anti-hypertensive medication, and 79.5% of medical institutions recommended medication. A total of 171 (81.4%) medical institutions were considering examinees’ sex, age, and accompanying diseases when they recommended anti-hypertensive medication to examinees. However, the majority of the screening institutions recommended medication, regardless of age, at least 150 mmHg levels of systolic BP.

**Table 4:** Prescription of anti-hypertensive drugs

	Yes		No		No response	
	n	(%)	n	(%)	n	(%)
Do you prescribe anti-hypertension drugs when a person has the following condition?						
SBP 140 mmHg, DBP 90 mmHg	83	(39.5)	120	(57.1)	7	(3.4)
SBP 150 mmHg, DBP 95 mmHg	167	(79.5)	35	(16.7)	8	(3.8)
SBP 160 mmHg, DBP 100 mmHg	184	(87.6)	18	(8.6)	8	(3.8)
SBP 140 mmHg, DBP 80 mmHg	47	(22.4)	154	(73.3)	9	(4.3)
SBP 160 mmHg, DBP 80 mmHg	147	(70.0)	54	(25.7)	9	(4.3)
When deciding whether to start anti-hypertensive drugs, do you consider the age of the patient?	171	(81.4)	39	(18.6)	0	(0.0)
Do you prescribe anti-hypertensive drugs when a person aged 65 years or older has the following condition?						
SBP 140 mmHg, DBP 90 mmHg	50	(29.2)	114	(66.7)	7	(4.1)
SBP 150 mmHg, DBP 95 mmHg	133	(77.8)	31	(18.1)	7	(4.1)
SBP 160 mmHg, DBP 100 mmHg	153	(89.5)	11	(6.4)	7	(4.1)
SBP 140 mmHg, DBP 80 mmHg	27	(15.8)	136	(79.5)	8	(4.7)
SBP 160 mmHg, DBP 80 mmHg	116	(67.8)	47	(27.5)	8	(4.7)

## Discussion

We evaluated the performance of hypertension screening and interpretation of BP measurements in medical institutions performing the national general health screening program in Korea. The JNC-8 and 2013 ESH/ESC as well as Korean Society of Hypertension recommend age-differential BP goal and treatment strategies [11, 17, 19]. The guideline for BP measurement and interpretation in Korea hypertension screening program is no different from these international guidelines.

Diagnosis of hypertension is commonly made from hypertension screening because hypertension has no specific symptoms. Hypertension can be diagnosed by noninvasive BP measurement and controlled by lifestyle changes or drugs, so it is a main target condition of national health screening. The benefits of screening for hypertension have been reported in previous studies. Systematic reviews support the effectiveness of BP screening of adults to detect hypertension and treat them to reduce cardiovascular disease [13]. A population-based cohort study in England reported that reductions in cardiovascular risk factor values were observed in people who regularly received the national health check-up [14]. A retrospective cohort study in Taiwan also reported that nationwide periodic health examinations was effective in promoting early treatment of hypertension, diabetes and hyperlipidemia in adults aged ≥40 years [15].

In Korea, the national health screening program controlled by government in nationwide level was launched in 1980. There are general screening program, national screening program for people at transitional age (44 and 66 years old), cancer screening, and infant screening program. Korea is investing more than 700 billion KRW in the national health examination, and many large private medical institutions are expanding their screening competitively in national screening area [20].

Several previous studies evaluated overall status of the national general health screening program, and suggested some points to be improved [20, 21]. However, there is no available study which specifically evaluated the performance of BP measurement and interpretation in the national general health screening program in Korea. Screening for hypertension is the most important and cost-effective component of the general health screening, but only limited data are available on specific condition and performance of hypertension screening. As we mentioned above, hypertension can be effectively detected and prevented through measurement of blood pressure, and proper treatment of high blood pressure can prevent cardiovascular events. However, if the quality of BP measurement is poor, interpretation of BP measurement is also not good and proper treatment is not possible. We hope that our report will be used as the baseline data for evaluating and improving the performance of national hypertension screening, because it is the first study on the actual status of BP measurement and interpretation of the national general health screening program.

The current study has some limitations. First, this study was a telephone survey that relies on self-report data and had a relatively small sample size. There is no assurance that the self-reported performance of hypertension screening and interpretation of BP measurements reflect real situation and an aspect of quality control. The self-reported data also may be influenced by the mode of administration of the questionnaire which can affect response rate or response contents [22]. Second, this study included medical institutions only in Seoul and Incheon areas, not in the whole country. Therefore, it may not be appropriate to generalize the present results to the entire. A third limitation is the potential for non-responder bias. It is possible that medical institutions with poor hypertension screening performance are less likely to participate in a telephone survey.

## Conclusions

In summary, the medical institutions performing the national general health screening program used mainly oscilloscopic devices for BP measurement, and most of them had measurement manuals and training protocols. Majority of the institutions measured BP multiple times with a resting period, and used the average values to determine an individual’s BP level. However, there is room for improvement in the equipment, environment and quality control procedures for BP measurement of the Korea national general health screening program.

## Abbreviations

BP: Blood pressure
KRW: Korean won
